# Boron: Functions and Approaches to Enhance Its Availability in Plants for Sustainable Agriculture

**DOI:** 10.3390/ijms19071856

**Published:** 2018-06-24

**Authors:** Fareeha Shireen, Muhammad Azher Nawaz, Chen Chen, Qikai Zhang, Zuhua Zheng, Hamza Sohail, Jingyu Sun, Haishun Cao, Yuan Huang, Zhilong Bie

**Affiliations:** 1 College of Horticulture and Forestry Sciences, Huazhong Agricultural University/Key Laboratory of Horticultural Plant Biology, Ministry of Education, Wuhan 430070, China; fareehamalik63@yahoo.com (F.S.); azher490@hotmail.com (M.A.N.); cuiyanhuashi@163.com (C.C.); zhangqk96@sina.com (Q.Z.); zuhzheng@163.com (Z.Z.); hamzadarbzu@gmail.com (H.S.); sunjingyu@webmail.hzau.edu.cn (J.S.); chsmugua@webmail.hzau.edu.cn (H.C.); huangyuan@mail.hzau.edu.cn (Y.H.); 2 Department of Horticulture, University College of Agriculture, University of Sargodha, Sargodha, Punjab 40100, Pakistan

**Keywords:** boron, ion uptake and transport, grafting, biostimulators, arbuscular mycorrhizal fungi, rhizobacteria

## Abstract

Boron (B) is an essential trace element required for the physiological functioning of higher plants. B deficiency is considered as a nutritional disorder that adversely affects the metabolism and growth of plants. B is involved in the structural and functional integrity of the cell wall and membranes, ion fluxes (H^+^, K^+^, PO_4_^3−^, Rb^+^, Ca^2+^) across the membranes, cell division and elongation, nitrogen and carbohydrate metabolism, sugar transport, cytoskeletal proteins, and plasmalemma-bound enzymes, nucleic acid, indoleacetic acid, polyamines, ascorbic acid, and phenol metabolism and transport. This review critically examines the functions of B in plants, deficiency symptoms, and the mechanism of B uptake and transport under limited B conditions. B deficiency can be mitigated by inorganic fertilizer supplementation, but the deleterious impact of frequent fertilizer application disrupts soil fertility and creates environmental pollution. Considering this, we have summarized the available information regarding alternative approaches, such as root structural modification, grafting, application of biostimulators (mycorrhizal fungi (MF) and rhizobacteria), and nanotechnology, that can be effectively utilized for B acquisition, leading to resource conservation. Additionally, we have discussed several new aspects, such as the combination of grafting or MF with nanotechnology, combined inoculation of arbuscular MF and rhizobacteria, melatonin application, and the use of natural and synthetic chelators, that possibly play a role in B uptake and translocation under B stress conditions.

## 1. Introduction

### 1.1. Functions of Boron (B)

B is one of the essential nutrients for the optimum growth, development, yield, and quality of crops [1]. It performs many important functions in plants and is mainly involved in cell wall synthesis and structural integration. According to a report, in tobacco (*Nicotiana tabacum* L.) and squash (*Cucurbita pepo* L.) plants, 95–98% of B is located in the cell walls of leaves [2]. B is cross-linked with pectin assembly, glycosylinositol phosphorylceramides (GIPCs), and rhamnoglacturonan-II (RG-II) [3,4] that control the tensile strength and porosity of the cell wall [5,6]. Limited B supply reduces RG-II dimer formation in pumpkin, resulting in abnormal (course and thick) cell wall formation [7]. B is involved in protein and enzymatic functioning of the cell membrane, leading to improved membrane integrity [1,8]. Optimum B concentration enhances the plasma membrane hyperpolarization, while B deficiency alters the membrane potential and reduces H^+^-ATPase activity [9]. Limited B availability reduces ATPase in plasmalemma-enriched vesicles of chickpea roots compared with control [10]. The direct effect of B on plasma membrane-bound proton-pumping ATPase influences ion flux: the alteration of H^+^, K^+^, PO_4_^3−^, Rb^+^, and Ca^2+^ ions across the membrane was observed in *Vicia faba* under B-deficient conditions [11].

The enhanced B requirement of young growing tissues proves its critical role primarily in cell division and elongation [12]. B starvation dramatically inhibits root elongation, with deformed flower and fruit formation due to impaired cell division in the meristematic region, whereas adequate B supply promotes advantageous root development [13]. B is involved in phenolic metabolism, and phenol accumulation is a typical feature of B-deficient plants [14]. B–sugar cis-diol complex formation is important to reducing phenol accumulation. However, plants fail to form this complex due to the shifting of the pathway from glycolysis to phosphate under B deficiency, resulting in phenolic compound production and accumulation [15]. B deficiency activates enzymatic and nonenzymatic oxidation by using phenol as substrate, resulting in elevated polyphenol oxidase and quinine concentrations, which are hazardous for plant growth and development [16]. B deficiency may trigger reactive oxygen species generation which drastically reduces ascorbic acid and glutathione metabolism [14]. Although B-deficient leaves of citrus showed antioxidant enzymatic activity against ascorbate, ascorbate peroxidase, and superoxide dismutase, they were not strong enough to protect against oxidative damage [17].

B plays a pivotal role in nitrogen (N) metabolism as it enhances nitrate levels and reduces nitrate reductase activity under limited B conditions [18]. A previous study has also highlighted the role of B in rhizobial N fixation, actinomycete symbiosis, and cyanophyceae heterocyst formation in leguminous crops [19]. Based on our knowledge, no study has investigated the direct involvement of B in photosynthesis. B deficiency affects photosynthesis indirectly by weakening vascular tissues responsible for ion transport [20]. Goldbach and Wimmer [9] suggested that the disruption in chloroplast membranes, stomatal apparatus, the energy gradient across the membrane, and thylakoid electron transport is a major reason for photosynthetic reduction under B-deficient conditions. During pollen tube growth and germination, B enhances the chances of fruit setting and improves seed production, leading to enhanced crop productivity. An adequate supply of B reduces the incidence of empty grains and enhances the yield by up to 5.5% in barley (*Hordeum vulgare* L.) [21], increases spike length and plant pigment content, hinders the chances of sterility in wheat (*Triticum aestivum* L.) [22], and improves the quality and shelf life of tomato (*Lycopersicon esculentum* L.) [23]. A previous study found that flowering and seed setting in *Arabidopsis thaliana* are maintained by over-expressing efflux B transporter *BOR1*, which not only increases the yield but also improves the mineral transport under B-deficient conditions [24].

**Figure 1:**
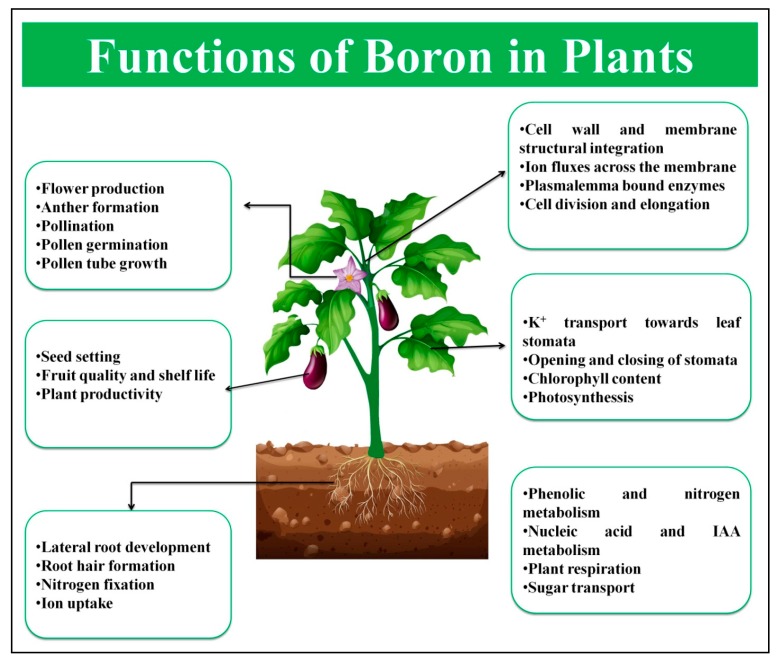
Functions of B in different parts of plant. IAA: Indoleacetic acid.

B influences the availability and uptake of other plant nutrients from the soil. An apparent increase in the uptake and translocation of P, N, K, Zn, Fe, and Cu in leaves, buds, and seeds was noticed after B application in cotton [25]. An increased or limited B supply decreases the nitrate levels by altering the nitrate transporter activity and inhibiting PMA2 transcript level in the roots, leading to decreased plasma membrane H^+^-ATPase activity [26]. However, Ca^2+^ channel (*CNGC19*) and Ca^2+^ transporter (ACA, CAX) genes are up-regulated under limited B conditions in *A. thaliana* roots, suggesting that B depletion results in the over-expression of the *CNGC19* Ca^2+^ influx channel in cells [27]. The functions of B in different parts of plants are summarized in Figure 1.

### 1.2. Deficiency Symptoms

Apart from the data obtained from agricultural reports that prove the involvement of B in plant growth and development, B often results in deficiency or toxicity because it is a unique micronutrient for which the threshold levels of deficiency and toxicity are very narrow [12]. B deficiency and excess are both widespread agricultural problems for higher plants in arid and semi-arid conditions. B deficiency was primarily observed in apples growing in Australia in the 1930s and subsequently reported in more than 132 field crops grown in sandy soils with low pH and organic matter from 80 different countries [28]. Depending on the age and species, plants manifest a wide range of deficiency symptoms, including stunted root growth, restricted apical meristem growth, brittle leaves, reduced chlorophyll content and photosynthetic activity, disruption in ion transport, increased phenolic and lignin contents, and reduced crop yield [1,8,20]. The prevalence of symptoms depends on the severity of the B-deficiency condition because plants show uniform deficiency symptoms on entire leaves but sometimes in the form of isolated patches. Given the immobile nature of B, it usually accumulates in mature leaves, whereas young leaves do not receive sufficient B for proper growth. Thus, the deficiency symptoms first appear on young leaves, including thick, curled, and brittle leaves with reduced leaf expansion; corky veins; interveinal chlorosis; yellow water-soaked spots on lamina; and a short internodal distance, resulting in a bushy plant appearance [14,29,30]. In severe cases, leaf apex necrosis and leaf dieback occur [12]. The expansion of stems and petioles leads to hollow stem disorder in broccoli and stem crack symptoms in celery [1]. However, in tomato, cauliflower, apple, and citrus, scaly surface development with internal and external corking of fruits is a typical feature associated with B deficiency [13,28].

Root elongation is restricted under B deficiency as cell division is limited [8,30]. Bohnsack and Albert [31] observed stunted root elongation of squash seedlings within 3 to 24 h under limited B supply, whereas seedlings attained normal growth rate within 12 h of B re-availability. B limitation negatively alters the reproductive performance of plants by causing abrupt changes in flowering and fruiting modes. This often results in empty and shriveled anthers, pollen tubes bursting, pollen viability loss, abscission of flower buds, failure of fruit setting, and premature fruit drop because of failure of photosynthate transport resulting in yield loss [12,14]. These findings suggest that B predominantly affects reproductive growth compared with vegetative growth in plants.

## 2. Mechanism of B Uptake and Long-Distance Transport under Limited Boron Supply

In the soil, B is found in the form of boric acid or borate; among all the essential elements, the percolation of B is in the form of uncharged molecules instead of ions [32]. B is considered as a highly lipid-bilayer-permeable [33] and intermediate mobile or phloem-immobile element depending on the membrane structure and plant species [14]. Initially, it has been argued that passive diffusion of uncharged boric acid across the lipid bilayer is the only mechanism of B transport from roots of vascular plants to aboveground plant parts such as stem and leaves [2]. This assumption was based on the high permeability of the lipid bilayer to boric acid, but Dordas and Brown [33] later revealed that the permeability of the lipid bilayer for boric acid is considerably lower than expected. At high B concentration, B is transported through passive diffusion, whereas active transport by the use of a special type of protein is achieved under low B supply [34]. To date, three pathways or mechanisms are recognized for the uptake and transport of B in plants: passive diffusion through plasma membrane; facilitated transport via channel proteins, such as the nodulin 26-like intrinsic proteins (NIPs); and high-affinity active transport reconciled by borate transporters (BOR) persuaded under low B availability [1,32,35].

### 2.1. Facilitated Transport by Channels

**Figure 2:**
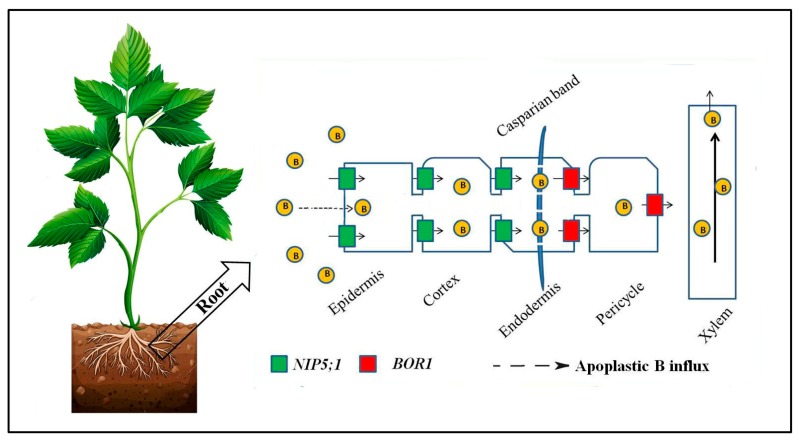
The mechanism of boron uptake and transport in plants under limited B conditions. Modified from Takano et al. [34].

Major intrinsic proteins (MIP) are reported as a channel for B transport. NIPs belong to the MIP subfamily and have the ability to transport small, uncharged molecules [34]. NIP5;1 was the first boric acid channel identified in *Arabidopsis thaliana* under limited B availability [36]. It is predominantly localized in the plasma membrane and is expressed in root epidermal, cortical, and endodermal cells. It increases B influx to root cells by enhancing the permeability of boric acid to the cell membrane. A 30% increase in B absorption under limited B supply was observed because of the up-regulation of maize PIP1 expression in *Xenopus laevis* oocytes [33]. NIP5;1 imports boric acid to the epidermal, cortical, and endodermal cells of roots but the Casparian band prevents the backflow of B [37]. B is extracted from soil solution via root cells, exported from xylem parenchyma cells into the stellar apoplasm, and then distributed in shoots and other plant parts under deficient B conditions (Figure 2). In *Arabidopsis*, AtNIP5;1 is the responsive channel for initial B uptake, whereas AtNIP6;1 is known to transport B to the growing plant parts under deficient B conditions [36,37]. Similarly, in rice, OsNIP3;1, a closest homolog to AtBOR1, regulates B distribution in shoots and enhances plant growth [38].

### 2.2. Active Transport by Transporters

The transport of B to other growing parts under B starvation is facilitated by a high-affinity transport system—BOR. Under limited B availability, this protein is essentially required for boric acid transport against the concentration gradient. BOR1 was identified in *Arabidopsis thaliana,* accumulating B in the pericycle cell plasma membrane under limited B availability [39,40]. BOR1 is expressed in pericycle cells of root stele and the expression of BOR1 in yeast decreased boric acid concentration in cells, proving its role as a borate transporter. The highly B-sensitive *Arabidopsis* mutant bor1-1 showed reduced growth and B accumulation particularly in shoots compared with roots [35]. Moreover, the maximum B concentration in xylem sap of wild-type *Arabidopsis* under limited B conditions suggested the role of BOR1 in xylem loading [39]. In rice, Nakagawa et al. [41] demonstrated the function of OsBOR1, a plasma-membrane-localized efflux transporter, in efficient B uptake as well as in xylem loading. BOR1 protein is generally localized in the plasma membrane, but at higher B concentration it is incorporated into the endodermis and then transported to vacuole for degradation, helping to avoid excessive B accumulation in plants at toxic levels of B availability [42]. BOR2 was found to be crucial for root elongation as well as for efficient RG-II cross-linkage formation in *Arabidopsis* under B-deficient conditions [43,44].

B tends to accumulate in mature leaves where its re-translocation to young tissues is restricted because of passive movement along the transpiration stream under high B soil conditions [35]. Conversely, its uniform distribution and re-translocation are reported in some plant species, such as apple, peach, plum, almond, olive, and tobacco, producing a significant amount of sugar alcohols, such as sorbitol and mannitol, utilized for phloem transport of photosynthates; this finding suggested that sugar alcohols are the principal factors conferring B phloem mobility due to the bonding of boric acid and sugar alcohols (*cis*-hydroxyl group) [45,46]. The transformation of a transgenic tobacco plant with sorbitol-synthesizing hormone enhanced B remobilization capacity through the phloem, increasing plant growth and yield by up to twofold compared with control plants under low B supply [47].

## 3. Approaches Utilized to Enhance B Uptake

Optimum plant performance depends on multiple factors including nutrient uptake capacity and distribution to other growing parts of the plant [1]. B is extensively distributed in the Earth’s crust and available in the form of uncharged boric acid or borate to plants depending on local soil conditions, such as soil moisture, soil temperature, soil pH, salinity, organic matter, and climatic conditions including rainfall [28]. The availability of B in many regions of the world, such as Brazil, the USA, China, Japan, and Korea, is limited because of its high solubility and leaching off by irrigation water or rainfall in shallow or coarse-textured soils [19,30]. Moreover, the chances of B availability under drought conditions or in soils with low organic matter content are also reduced because of the alkalization and breakdown of organic matter, respectively [28]. The maintenance of optimal B concentrations in the soil solution is crucial for maximum production, which can be achieved by implementing several beneficial and eco-friendly techniques. These techniques not only enhance the B uptake and transport to other plant parts but also improve the soil fertility and crop production. Some important approaches to enhance the B acquisition are described as follows.

### 3.1. Modification of Root Traits

The soil supplies a large quantity of ions for plants’ optimum growth and productivity. Plants have evolved several adaptive mechanisms in response to differences of ion availability under the prevailing rhizospheric conditions. These adaptive functions are performed by the plant root system, which not only absorbs water and nutrients from the soil but also provides anchorage to the plant. Root morphological traits are highly important to soil probation and efficient resource utilization in plants for better survival, particularly under suboptimal ionic conditions [48]. Root traits vary greatly for different plant species, soil structures, and ion availabilities. The roots normally absorb 30% of total water and 10% of nitrates from the soil solution [14]. Maximum absorption of nutrients in plants is achieved by changing the postembryonic development of the root system by modifying the dynamic root structure, including root meristematic division; lateral root and root hair formation; and increased root length, diameter, and surface area, which mediates its adaptation to low nutrient availability [49,50]. Increased root length and dense root hairs improve root system exploratory capacity by enabling plants to capture nutrients deeper in the root zone that are usually distributed widely and unevenly in the soil under limited ion conditions. Certainly, there is a significant relationship between B uptake and root morphology [32]. B limitation inhibits the primary root development leading to an excessive number of lateral roots with new root hair formation in an area closer to the root meristem, which enhances B uptake capacity [51]. Under limited ion conditions, the root architecture is significantly altered due to root sucrose and amino acid production and soil pH reduction [50]. Meanwhile, plants from families Proteaceae, Fabaceae, Cyperaceae, Cucurbitaceae, and Myricaceae start to develop cluster roots [52] with dense root hairs, suggesting it a competitive advantage for plants regarding nutrient uptake grown under an ion-stress-induced environment.

In different plant species, the uptake of ions is specific to root sections and categories. For instance, in wheat, lateral roots absorb and transport more water with maximum ion absorption compared with nodal roots [53]. Similarly, fine roots are more reliable in water and ion uptake compared with the coarse and middle roots of *Gossypium hirsutum* [49]. The nutrient-absorbing capabilities can be triggered by the use of plant growth regulators, particularly auxin or ethylene, which are implicated in cell epidermal formation, nutrient deficiency response regulation, and root architecture modification. The interaction between auxin and ethylene is of prime importance in deciding the fate of root elongation by regulating the gene expression of hormone synthesis and translocation [54]. Martin-Rejano et al. [51] investigated the effect of low B availability on two *Arabidopsis* mutants—*ein2-1* and *aux1-22*—and found twofold less primary root inhibition with a greater number of root hairs in the *aux1-22* mutant compared with *ein2-1* and control, suggesting the involvement of ethylene in root hair formation under low B supply through the overexpression of ethylene reporter EBS::GUS and ACS11::GUS in the primary root maturation zone. The application of auxin significantly alters root morphological traits of *A. thaliana* (*axr2* mutant) grown under low and high phosphorus conditions, showing maximum root hair elongation of 0.9 and 0.3 mm, respectively. Furthermore, exposure of the *axr2* mutant grown in high-phosphorus medium (with less root hairs) to indoleacetic acid (IAA) encourages root elongation with denser root hair formation [55] by promoting AUX/IAA repressor degradation due to the interaction of *SCF^T1R1/AFB1-3^* ubiquitin ligase and AUX/IAA protein, which regulates the expression of auxin responsive factor [56]. Similarly, ethylene and auxin mitigate chlorosis by transferring cells in root rhizodermis, sub-apical root swelling, and enhancing root hair formation that boosts the ion uptake capacity of sunflower roots [57].

On the basis of the phenotypic variation of crops for ion absorption, the breeding of crops for efficient nutrient acquisition by modifying their root traits has been performed via quantitative trait loci (QTLs), which help to identify the genetic markers and genetic inheritance of nutrient-related traits [58]. Moreover, the exploitation of some other novel approaches, such as the use of natural or synthetic chelators, phytosiderophores, diethylenetriamine pentaacetic acid, ethylenediaminetetraacetic acid, and melatonin, also enhances mineral uptake via modifying the root architecture and translocation to aerial plant parts [59,60,61,62]. However, the QTL mapping and the use of these substances require further investigation for B uptake and translocation, particularly under B deficiency. The application of melatonin enhances the N concentration in the roots of watermelon seedlings grown under low N conditions by enhancing the root elongation, surface area, and root diameter [61]. However, in the case of B, more research is needed to determine whether a similar mechanism exists.

### 3.2. Grafting

The limited availability of B to plants can be mitigated by incorporating B in the soil or foliar application of B-containing fertilizers. However, this increases the cost of crop cultivation and may cause B toxicity due to the narrow range from deficiency to toxicity. Another appropriate and environmentally friendly technique to reduce this problem is the use of suitable rootstocks for different crops [30,63,64] that can absorb a large quantity of B from the soil and transport it to upper plant parts to be utilized for proper physiological functioning. Indeed, rootstocks affect plant nutritional status in various crops due to their efficacious water- and mineral-absorbing capabilities from soil solution compared with self-rooted plants [65,66,67,68,69]. Additionally, rootstocks enhance the tolerance of scion cultivars to B deficiency [70] and toxicity [71]. Grafting increases water and ion uptake by modifying the vascular bundle connection between the scion and rootstock allowing enhanced synthesis of carbohydrates and thereby improving plant growth [72]. Initially, the influence of rootstocks on mineral acquisition was hypothesized to be attributed to physical characteristics of roots, but later on, the genetic makeup of rootstocks and vigor of the scion were identified to be responsible for ion uptake and translocation [73]. The physiological interactions of scion and rootstock and their influence on mineral acquisition have been extensively studied in plant species. For citrus, Carrizo citrange (*Citrus sinensis* Osb. × *Poncirus trifoliata* [L.] Raf.) and red tangerine (*C. tangerina*) are genetically efficient, trifoliate orange (*P. trifoliata* [L.] Raf.) is moderate, whereas sour orange (*C. aurantium* L.) and fragrant citrus (*C. medica*) are inefficient rootstocks for B uptake and transport to the plant canopy under limited B availability [63]. Liu et al. [74,75] evaluated the effect of B on Carrizo citrange (*C. sinensis* Osb. × *P. trifoliata* [L.] Raf.) and trifoliate orange (*P. trifoliata* [L.] Raf.) rootstock grafted on orange plants. An increase in B uptake and newly absorbed B concentration in lower and upper leaves of Carrizo citrange grafted plants was observed compared with trifoliate-orange-grafted plants. In pistachio (*Pistacia vera* cv. Kerman), *P. atlantica* rootstock greatly absorbed B and other nutrients from the soil solution with maximum concentration (1.2–2.4 times more) in leaves followed by PG-II compared with other rootstocks [76].

Notably, the specific nutrient-absorbing pattern depends on the genotype of the rootstock. For example, trifoliate orange (*P. trifoliata*), Carrizo citrange (*C. sinensis* Osb. × *P. trifoliata* [L.] Raf.), and mandarins (*C. reticulata* Blanco) are efficient citrus rootstocks for the absorption of N, P, K, Mg, and B, whereas Hill sweet oranges (*C. sinensis*) are specified for Ca, Na, Mn, and Zn uptake from the soil solution [77]. While working on different cherry rootstocks, Hrotko et al. [78] observed different rootstock-dependent macro- and micronutrient absorption patterns. GiSelA6 (*Prunus cerasus* × *P. canescens*, Gi 148/1) rootstock absorbed a large N, P, K, Zn, Mn, and B concentration from soil solution with Mg, Ca, and Cu deficiencies. Similarly, *P. mahaleb* provided adequate N, P, K, Ca, Mg, and Fe to the leaves, but roots tend to develop Zn, Mn, and B deficiencies. 

The distribution of ions in different plant parts also varies greatly, depending on the availability of ions in the soil solution. In Newhall orange (*C. sinensis* Osb.), Sheng et al. [29] observed decreased B content in leaf (23–53%) and scion (40–65%) tissues but increased in rootstock parts (35–60%) grafted onto Carrizo citrange (*C. sinensis* Osb. × *P. trifoliata* [L.] Raf.) compared with trifoliate orange (*P. trifoliata* [L.] Raf.) when exposed to limited B supply. Similarly, in pears, with the use of *Prunus* rootstock, El-Motaium et al. [71] measured B accumulation in the roots, stems, and leaves and observed no remarkable increase in root tissues. However, a strong correlation between rootstock and B uptake was observed in the leaves and stems with an increase of B concentration by up to 50–80% and 100–300%, respectively. Wang et al. [79] studied the B absorption pattern in four citrus rootstock–scion combinations and observed a maximum B concentration in the buds and leaves of Fengjie-72 navel orange (*C. sinensis* [L.] Osb. cv. Fengjie-72) grafted on Carrizo citrange and trifoliate orange plants under inadequate B supply. However, B accumulation in Newhall scion grafted onto Carrizo citrange was higher (24%) compared with other combinations. Moreover, a higher ratio of available B (36%) was found in the leaves of Carrizo citrange compared with trifoliate-orange-grafted plants. Details of increased B concentration (%) in different plant parts through grafting are summarized in Table 1.

The role of B for the uptake of other ions is also reported in some studies. Zhou et al. [30] compared seven different rootstocks of citrus under limited B supply and found a decrease in leaf B concentration from 83.6% to 72.7% in all seedlings, except for Carrizo citrange (45.8%), suggesting that Carrizo citrange is an efficient rootstock for B uptake. Moreover, a range of macro- and micronutrient absorption patterns were also observed. For example, decreased Ca, K, Mg, and Zn content and increased Fe, and Mn concentration were observed in the root and leaf tissues of the scion. Wang et al. [80] practiced the inarching of Carrizo citrange (*C. sinensis* Osb. × *P. trifoliata* [L.] Raf.) on Newhall orange (*C. sinensis* Osb.) budded onto trifoliate orange (*P. trifoliata* [L.] Raf.) and found that plants sustained better growth under limited B availability in root medium and responded positively to an enhanced B level in new leaves, twigs, scion, and rootstock stem, proving that inarching is an excellent technique for ion uptake from the soil solution. Thus, this technique may be utilized to address the B deficiency of citrus plants already growing in orchards.

### 3.3. Biostimulators

Another technique that can be utilized to enhance mineral acquisition with low input is the use of biostimulators. In the last few decades, biostimulators have played an important role in physiological modifications of plants to optimize plant production. Biostimulants are materials other than fertilizers, soil improvers, or pesticides that influence plant metabolic processes, including cell division, respiration, photosynthesis, and ion uptake, when applied in a small quantity [81]. They interact with the plant signaling cascade to reduce negative plant reactions under stress conditions, leading to optimum plant production [82]. Recently, the application of organic biostimulators has received considerable attention from researchers because of their multidirectional benefits. Their role toward improved soil structure, seed germination, crop quality, and yield by reducing abiotic stresses has been well documented [83]. Moreover, several studies have highlighted the involvement of biostimulators in macro- and micronutrient acquisition and translocation [84,85]. Biostimulated crops are less sensitive to stressful environmental conditions and more efficient for ion uptake under limited ion conditions because of improved antioxidant production [83]. Among organic biostimulants, humic substances (HS) are well known for improving soil structure and root architecture by enhancing root H^+^-ATPase activity; therefore, they are extensively utilized for ion acquisition depending on the concentration, plant species, and environmental conditions [84]. The field trials of biostimulant soil application on *Vicia faba* cv. Giza 3 beans showed an improved soil architecture and ion uptake compared with control [86]. Conversely, the composted sewage sludge from HS improved the growth and yield of *Capsicum annuum* L. cv. Piquillo. These effects were associated with increased micronutrient availability to the substrate [87] and improved microbial activity within plants, which help to prevent ion leaching by decreasing soil pH through producing organic acids (citrate, oxalate, malate). The HS forms a complex with micronutrients and the plasma membrane generates the proton motive force to aid active and passive transport of ions through the symplastic pathway, thus enhancing trace element availability to plants [88].

Amino acids improve plant nutrition by affecting soil microbial activity through the production of a beneficial microbial community and nutrient mineralization in the soil solution, thus enhancing micronutrient mobility [84]. Seaweed extract contains several ions, growth regulators, carbohydrates, proteins, vitamins, and polyuronides, including alginates and fucoidans. These polyuronides can form highly cross-linked polymers and condition the soil, thereby improving the water retention and ion uptake capacity within the soil [89]. Kahydrin, a commercial seaweed component, acidifies the rhizosphere by altering the plasma membrane proton pump and secretes H^+^ ions that change the soil redox condition and make the metal ions available to plants, leading to improved crop production [90]. Turan and Kose [91] applied three seaweed extracts, including Maxicrop, Algipower, and Proton, on grapevine (*Vitis vinifera* L. cv. Karaerik) to check the ion uptake efficacy under optimal and deficient ion availability. Maximum micronutrient uptake under optimal conditions were observed with no significant difference among the three kinds of extracts. The alteration in uptake of one ion influences the availability of another ion [85], supporting the idea of B uptake through biostimulator application, but this requires further investigation.

The application of biofertilizers opens new routes of ion acquisition by increasing nutrient use efficiency in plants. In this regard, mycorrhizal and non-mycorrhizal fungi, endosymbiotic bacteria, and plant-growth-promoting rhizobacteria are important because of their dual function as microbial biostimulants and biocontrol agents. We explain the functions of these biostimulators and their possible relationship with ion acquisition in plants.

#### 3.3.1. Mycorrhizal Fungi (MF)

Microorganisms such as bacteria and fungi present in the soil create a symbiotic relationship with roots of higher plants by playing a crucial role in N fixation, chitinase production (toxic-to-root pathogen), ion acquisition, and increasing plant fitness under contradictory ecological surroundings [92]. Fungi that create associations with plant roots are known as mycorrhizas; among them, arbuscular MF (AMF) are tremendously important in soil fertility and nutrient acquisition of plants [93]. These ectomycorrhizas are naturally widespread in the terrestrial ecosystem and are associated with more than 80% of vascular plants, except for a few members of the families Chenopodiaceae, Proteaceae, Cyperaceae, and Cruciferae [94]. This affiliation between two symbiotic partners is based on bidirectional returns and, under adverse conditions, MF capture soil nutrients and act as transporters of these nutrients from soil to plant roots by acquiring plant sugars in return for their metabolism [95]. For example, in the Arctic tundra, AMF contribute 61% to 86% of N absorption and transport to plants and acquire 8% to 17% of plant photosynthetic carbon in return [96]. The nutrient-absorbing ability and retaining or excluding capacity are because of the fungal mycelium that creates a hyphal sheath around the fine roots of vascular plants or hyphal spirals within root cortical cells; these structures allow the beneficial plant nutrients to enter and prevent the uptake of heavy metals, such as cadmium, chromium, lead, and arsenic, from the soil solutions, thereby protecting host plants from abiotic stresses [97]. Sarkar et al. [93] observed an increase in total ion concentration of macro- and micronutrients in root, stem, and leaf of *Miscanthus* *sacchariflorus* plants inoculated with AMF grown under natural sterilized soil conditions. The availability of these minerals to plants increased because of the action of organic compounds released from the plant roots and fungi.

The B concentration in plants is affected by AMF inoculation. Some studies report reduced [98], unaffected [99], and enhanced [100] acquisition of B in shoots of MF-inoculated plants, but the exact role of mycorrhizal functioning for B has not been proven yet and requires further investigation. The evolutionary vascular structure of higher plants demonstrates the role of B in lignification [101]. The passive uptake of boric acid appears to occur in plants, but the mechanism of mycorrhizal B uptake is still poorly understood. The crucial involvement of sugar alcohols (sucrose, sorbitol, and mannitol) in B remobilization within plant tissues is widely documented [45,46]. According to Lewis’s [101] hypothesis, sucrose is a major carbohydrate that is mainly responsible for B mobilization because of its low B affinity in vascular plants. By contrast, fungal carbohydrates, particularly mannitol, have high affinity to readily form a complex with B, resulting in poor B mobility from fungal symbiotic partner to the host. However, this mannitol–B complex mobility has been observed in some mycelia, which allows the continuous uptake and long-distance transport of B in plants [102].

The considerable B accumulation in plant parts is proven by the presence of several mycorrhizal and saprotrophic species (Table 1). The concentration of B in the presence of *Amanita muscaria* was merely 0.01 mg Kg^−1^, whereas it was increased by up to 280 mg Kg^−1^ in the presence of *Paxillus involutus*, thereby showing the potential of MF in B acquisition [95]. This difference may be attributed to the usage of water and carbohydrates by different species of MF for long-distance transportation or by different soil conditions. Under acidic and alkaline conditions, *Glomus intraradices* inoculated maize plants translocate higher amounts of B to shoots compared with *G. etunicatum* and *G. diaphanum* inoculated plants [108]. However, the acquisition was more pronounced in acidic soils compared with alkaline soils because high pH stabilizes the mannitol–B complex and prevents B from moving into plant cells. The substantial B accumulation in the mycelium–root system and the transient B–mycorrhizal bonding affect the B translocation from mycorrhiza to shoot. Ruuhola and Lehto [106] inoculated silver birch (*Betula pendula*) seedlings with two species of ectomycorrhizal fungi—*Paxillus involutus* and *Laccaria* sp.—to estimate the contribution of MF toward B uptake and translocation. They found 30–40% ectomycorrhizal colonization and higher net B uptake initially in roots and then in stem and leaves in *Laccaria* inoculated plants because of B retention at the early symbiotic phase compared with other species and non-inoculated plants. Similarly, *P. involutus* inoculated plants showed enhanced acquisition and then translocation to the host plant stem and leaves during a ^10^B labeling study in *B. pendula* with initial B retention in mycorrhizal roots for 72 h [105]. Meanwhile, in rough lemon (*Citrus jambhiri* Lush), the foliar and soil amendment of B inoculated with *Glomus fasciculatum* not only increased total B accumulation in the leaves by up to 11–18% but also enhanced the exudation of root sugars and amino acids, compared with non-inoculated plants [107].

Besides B mineral acquisition, AMF also increase the uptake of other nutrients, resulting in improved plant growth. In the case of shortleaf pine seedlings (*Pinus echinata* Mill), the inoculation of *Pisolithus tinctorius* enhanced the P, K, Mg, B, Cu, and Mn content by providing a greater absorptive root surface area with maximum mycorrhizal colonization within the roots, thus limiting water and nutrient depletion in the root zone area [109]. Additionally, improvements in plant development, including increased stem height and stem weight, were observed in AMF-inoculated plants compared with the non-inoculated plants. Although a number of studies have shown that the application of MF could be a meaningful approach toward B acquisition and translocation within plants, there are still certain aspects that need further investigation. For instance, the molecular mechanism of B uptake by fungi would help to improve understanding of how MF inoculation improves B uptake and translocation, thereby reducing the requirement of B fertilizers for crop production on a sustainable basis. Furthermore, whether AMF inoculation might affect the activity or efficiency of B transporters requires investigation.

#### 3.3.2. Plant-Growth-Promoting Rhizobacteria (PGPR)

Rhizobacteria, often referred to as plant-growth-promoting rhizobacteria, are agronomically useful and active root-colonizing microbes that form a symbiotic association with plant roots. They are involved in N fixation [110], salinity and drought tolerance [111], enzyme production against pathogenic microorganisms, nutrient solubilization, and phytohormone production (IAA, cytokinins, and gibberellins), which promote root proliferation [112], resulting in ample water and nutrient uptake. In lentil (*Lens culinaris* Medik), PGPR inoculation not only enhanced the N (2.26–2.95%) and P (0.52–0.82%) acquisition in root, stem, and grain but also improved plant growth in terms of root and shoot length, and fresh and dry weights. The IAA contents are high in PGPR strains LCA 1 and LCA 5 [113], suggesting that IAA production varies in different species due to several biosynthetic pathways, gene regulatory mechanisms, and enzyme production to convert IAA into its conjugates [114]. In a previous report, improvement in phytohormones (IAA, GA_3_), macronutrients, and micronutrients was observed by applying PGPR in *Raphanus sativus* and *Mussa* spp. [115,116]. The P content in potato (*Solanum tuberosum* L.) was improved by 43.1% with the inoculation of *Bacillus cereus* P31 strain, whereas *Achromobacter xylosoxidans* strain P35 increased the N and K content by up to 50.5% and 48.3%, respectively [117].

The role of bacteria in absorbing excessive levels of B from soil solution has been documented. Several B-tolerant bacterial strains of *Bacillus*, *Chimaereicella*, *Gracilibacillus*, *Lysinibacillus*, *Boronitolerans*, *Variovorax*, *Pseudomonas*, *Mycobacterium*, and *Rhodococcus* absorb toxic levels of B from soil [118,119,120]. Furthermore, several species of PGPR have proved helpful in increasing mineral acquisition (N, P, K, Ca, Mg, Fe, and Zn) and improving plant growth by enhancing root length and modifying root structure through effective colonization, thereby increasing the fresh and dry weights of roots and shoots, chlorophyll and protein contents [112,117], photosynthetic rate [121], and yield of several horticultural [122,123] and agronomic crops [124,125]. However, there is still no evidence available that explains the role of PGPR in B uptake and utilization efficiency. Thus, further investigation is required to determine efficient B-capturing bacteria that may enhance B availability to crops under limited B supply.

### 3.4. Nanotechnology

With limited availability of nutrients and water resources, optimum agricultural growth can be achieved by improved crop production practices supported with effective use of modern technology. Nanotechnology is a novel and emerging approach that can be utilized in the agriculture sector for biotic and abiotic stress management, disease detection, and nutrient absorption [126,127]. It improves the production and nutrient utilization efficiency of plants by consuming a small quantity of resources compared with conventional approaches. Nanoparticles (NPs) can boost plant metabolism because of their unique physiochemical properties, and, hence, enhance crop yield and nutritional value [128]. For instance, the application of copper NPs in watermelon improved plant growth and development compared with control [129]. Similarly, zeolites and hydrogels are reported to absorb environmental contaminants and improve soil water holding capacity [130].

Micronutrients such as Cu, Fe, B, Mn, Zn, Cl, and Mo are being progressively depleted from the soil solution because of steady crop production. Chemical fertilizers are used to solve this problem to obtain better plant growth but, due to leaching properties and deleterious environmental impacts, there is a need to minimize the nutrient losses in fertilization. Recently, the concept of nano-fertilizer has been gaining popularity because of its slow-release and minimum leaching properties [126]. Chitosan NPs efficiently reduced fertilizer consumption and environmental pollution [126]. A combination of superabsorbent polymer with slow-release fertilizers significantly improved crop nutrition and yield by reducing ion and water loss [131]. Foliar or soil application of micronutrient nano-formulated fertilizers may be used to improve soil health with maximum nutrient uptake that will ultimately improve plant growth. According to a report, application of CeO_2_ and ZnO NPs did not enhance the macronutrient concentration in *Cucumis sativus* fruit. However, these NPs changed the fruit profile of micronutrients by obviously increasing their concentration in fruits [132]. Similarly, nano-materials such as multiwalled carbon nanotubes (MWCNTs) have been reported for their incredible ability to improve the growth of *Zea mays* by increasing water and micronutrient uptake efficiency [133]. Nano-titanium dioxide (TiO_2_) application promoted chlorophyll synthesis and photosynthetic activity by increasing the ion uptake efficiency of spinach [134]. Considering this, nanotechnology may be used to enhance the B uptake and utilization efficiency of plants, resulting in reduced B fertilizer requirements for agricultural use.

## 4. Future Perspectives

The abilities of crops to efficiently utilize B resources vary considerably. Thus, from an agricultural point of view, there is a need to identify the important cultivars of agronomic and horticultural crops with vigorous root systems to utilize the available B and that can thrive best under B shortage. For example, in cucumber, the Ashlay variety performs well under Fe-deficient conditions with high nutrient and chlorophyll content and reduced chlorosis [135]. In rape seed, the B use efficiency is closely related to sugar production [136], earlier flowering, and bolting [137], which certainly reduce the B requirement for vegetative biomass production. Knowledge of the rootstock and scion relationship may be helpful in identifying excellent root systems of crops that are tolerant to deficient or toxic B conditions. Furthermore, the mechanistic investigation at the molecular level for B in plants opens new prospects to improve B stress tolerance in crops.

Indeed, grafting and AMF inoculation improve plant physiological and nutritional aspects and a number of studies have proved their pivotal role in B uptake [74,75,79,105]. Additionally, nanotechnology is an emerging technique to solve plant-nutrition-related problems. The combination of these techniques may improve B uptake. For instance, a combination of grafting and Cu NPs improved growth and development of watermelon by increasing ion uptake [129]. Melatonin application improves plant performance by inducing resistance against stress conditions. According to a report, melatonin application reversed the toxic effect of B by moderating B accumulation in leaf and fruit, increasing photosynthetic activity, and improving dry weight that ultimately enhanced plant growth of *Capsicum annuum* [138]. Similarly, in watermelon, melatonin application enhanced the N concentration in roots by improving root elongation, root diameter, and root surface area under limited N availability [61]. However, no evidence for B uptake under deficient conditions has been found yet, and that requires further investigation.

A stressful environment exerts a negative impact on plant growth and development. However, this stress can be mitigated by the use of PGPR and AMF. Most studies on MF and rhizobacteria applications focused on improving B acquisition and plant growth under normal and stress conditions [100,105,106]. Certainly, these applications enhance the water and B content of plants, but the dual inoculation of both MF and PGPR could be more useful in B assimilation compared with their individual use. In some plant species, the combined inoculation of MF and PGPR improves growth by enhancing water and macronutrient contents [139]. However, to the best of our knowledge, no data are available for micronutrients, particularly B. The mechanism of nutrient acquisition by these microorganisms is also poorly understood. Therefore, the role of combined inoculation of these microorganisms for efficient B acquisition and its molecular mechanism need to be investigated further to obtain better outcomes and to improve B uptake and utilization in plants.

## Figures and Tables

**Table 1:** Influence of grafting and mycorrhizal fungi on B uptake of plants.

**Grafting**				
**Crops**	**Rootstocks**	**Plant Parts Utilized**	**Increase in B Concentration (%)**	**References**
Navel orange (*Citrus sinensis* Osb)	Carrizo Citrange [*Citrus sinensis* (L.) Osb. × *Poncirus trifoliata* (L.) Raf.]	New leaves Middle leaves Basal leaves Roots New twig Old twig	24–51 53 66–149 63 25 24	[29,73,79]
	Trifoliate orange [*Poncirus trifoliata* (L.) Raf.]	New leaves Roots	6 12	[74]
	Troyer citrange (*Citrus sinensis* Washington × *Poncirus trifoliata*)	Leaves	34	[76]
	Cleoptra mandarin (*Citrus reticulata*)	Leaves	29	[76]
	Rough lemon (*Citrus Jambhiri* Lush)	Leaves	29	[76]
Sweet Cherry *(Prunus avium* L.) cultivar Petrus	GiSelA 6 (*Prunus cerasus* × *Prunus canescens*, Gi 148/1)	Leaves	20	[77]
	Sweet Cherry *(Prunus avium)*	Leaves	15	[77]
	Magyar	Leaves	14	[77]
Sweet Cherry *(Prunus avium* L.), cultivar Rita	GiSelA 6 (*Prunus cerasus* × *Prunus canescens*, Gi 148/1)	Leaves	13	[77]
	Sweet Cherry (*Prunus avium*)	Leaves	8	[77]
Grapes (*Vitis vinifera* L.)	Riparia Gloirede Montpellier (RGM) and 1103 Paulsen (*Vitis vinifera* L.)	Leaves	93	[103]
Pistachio (*Pistacia atlantica* L.) cultivar Kerman	*Pistacia atlantica*	Leaves	19	[47]
	*Pistacia integerrima*	Leaves	3	[47]
	UCBI (*Pistacia atlantica× Pistacia integerrima*)	Leaves	68	[47]
Tomato (*Solanum lycopersicum* L.)	Maxifort (*Solanum lycopersicum* L. × *Solanum habrochaites* S. Knappand D.M. Spooner)	Leaves	3	[104]
	**Mycorrhizal Fungi**			
**Crop**	**AMF Species**	**Plant Part Utilized**	**Increase in B Concentration (%)**	**Reference**
Silver Birch (*Betula pendula*)	*Laccaria* sp.	Roots Stem Leaves	5–19 5 3	[105,106]
	*Paxillus involutus*	Roots Stem Leaves	78 5–15 14	[105,106]
Rough lemon (*Citrus Jambhiri* Lush)	*Glomus fasciculatum*	Leaves	11–18 *	[107]
Maize (*Zea mays* L.)	*Glomus etunicatum* WV579A	Shoot	331–689	[108]
	*Glomus diaphanum* WV579B	Shoot	261–510	[108]
	*Glomus intraradices* WV894	Shoot	284–531	[108]

* Boron accumulation in the leaves; AMF: arbuscular mycorrhizal fungi.

## Abbreviations

ACA	Auto-inhibited Ca^2+^-ATPase
AMF	Arbuscular mycorrhizal fungi
CAX	Cation exchanger
CNGC	Cyclic nucleotide-gated ion channel
GA3	Gibberellic acid
GIPC	Glycosylinositol phosphorylceramides
HS	Humic substances
IAA	Indolacetic acid
MF	Mycorrhizal fungi
MWCNTs	Multiwalled carbon nanotubes
NIP	NOD-26-like intrinsic protein
PGPR	Plant-growth-promoting rhizobacteria
RG-II	Rhamnoglacturonan-II
